# Association of the CHADS_2_ and CHA_2_DS_2_-VASc scores with left atrial enlargement: a prospective cohort study of unselected atrial fibrillation patients

**DOI:** 10.1007/s11239-014-1154-6

**Published:** 2014-12-10

**Authors:** Anna Hrynkiewicz-Szymanska, Miroslaw Dluzniewski, Anna E. Platek, Filip M. Szymanski, Joanna Syska-Suminska, Agnieszka Klos-Szadryn, Marta Glinka, Malgorzata Strojek, Alicja Kuciej, Monika Tomaszewska-Kiecana

**Affiliations:** 1 Department of Cardiology, Hypertension and Internal Diseases, Medical University of Warsaw, Kondratowicza 8 Street, 03-242 Warsaw, Poland; 2 1st Department of Cardiology, Medical University of Warsaw, 1A Banacha Street, 02-097 Warsaw, Poland

**Keywords:** Atrial fibrillation, Left atrial enlargement, Stroke risk, CHADS_2_, CHA_2_DS_2_-VASc

## Abstract

Assessment of thromboembolic risk is crucial for proper management of atrial fibrillation (AF) patients. Currently used risk score base only on scarce clinical data and do not take into consideration parameters including echocardiographic findings. The aim of this study was to evaluate if left atrium (LA) enlargement is associated with higher thromboembolic risk assessed by CHADS_2_ and CHA_2_DS_2_-VASc scores in a cohort of unselected non-valvular AF patients. Data from 582 AF hospitalizations occurring between November 2012 and January 2014 were analyzed. All patients underwent a standard transthoracic echocardiography and had their thromboembolic risk assessed in both CHADS_2_ and CHA_2_DS_2_-VASc scores. In 494 enrolled patients (48.5 % male; mean age 73.4 ± 11.5 years) AF was classified as paroxysmal in 233 (47.3 %), as persistent in 109 (22.1 %), and as permanent in 151 (30.6 %) patients. LA was enlarged in 426 (86.2 %) patients. Enlargement was classified as mild in 99 (20.0 %) patients, as moderate in 130 (26.3 %) patients, and as severe in 196 (39.7 %) patients. Patients with enlarged LA had higher mean CHADS_2_ score (2.0 ± 1.5 vs. 2.6 ± 1.3; *p* = 0.0005) and CHA_2_DS_2_-VASc (3.8 ± 2.0 vs. 4.4 ± 1.8; *p* = 0.02) score than patients with normal LA. The both mean scores rose along with rising LA diameter. LA enlargement is highly prevalent in AF patients. Higher thromboembolic risk assessed by both CHADS_2_ and CHA_2_DS_2_-VASc scores is associated with presence of LA enlargement. Echocardiographically assessed LA size may be an additional parameter useful in thromboembolic risk stratification of AF patients.

## Introduction

Assessment of thromboembolic risk is crucial for proper management of atrial fibrillation (AF) patients and prevention of one of its most important complications—an cardioembolic stroke. AF is directly responsible for occurrence of approximately 1 out of every 5 strokes [1]. Therefore current guidelines recommend anticoagulation therapy in patients with elevated stroke risk [2, 3]. Tools recommended for the stroke risk assessment are the CHADS_2_ and CHA_2_DS_2_-VASc scores. Those are point scores, which calculate the annual stroke risk basing on clinical parameters like presence of comorbidities including age, congestive heart failure, diabetes mellitus or history of stroke [4, 5]. Unfortunately, as previously reported for the case of obstructive sleep apnea, the CHADS_2_ and CHA_2_DS_2_-VASc, like many point scores, are likely to omit many important parameters associated with elevated thromboembolic risk [6].

One of the stroke risk factors overlooked by the scores is the size of left atrium (LA). AF-associated thrombi, which are responsible for stroke, are forming mostly in the LA and its appendage. LA size and its morphology were previously shown to be predictive of thromboembolic risk [7, 8]. Therefore, on one side presence of the enlarged atrium is associated with greater thromboembolic risk, and worsens the outcome of invasive strategies for sinus rhythm restoration [9]. On the other hand, in many cases LA enlargement is partially caused by the AF-related hemodynamic disturbances or comorbidities highly prevalent in AF patients, and it was shown that LA size tends to decrease when sinus rhythm is restored [10]. The mutual exacerbation of AF and LA enlargement often coexist in patients, but LA size was not implemented into the currently used risk assessment schemes.

The aim of this study was to evaluate if LA enlargement is associated with higher thromboembolic risk assessed by CHADS_2_ and CHA_2_DS_2_-VASc scores in a cohort of unselected non-valvular AF patients.

## Methods

### Study population

The study was designed and conducted with the accordance of the Declaration of Helsinki and it was approved by the Regional Ethics Committee. We prospectively analyzed data on continuous hospitalizations for AF that occurred between November 2012 and January 2014 in a tertiary University Hospital Cardiology Department. Inclusion criteria were age ≥18 years, primary diagnosis of AF, echocardiographic evaluation underwent during current hospitalization. We excluded patients with valvular AF, fatal condition with estimated life expectancy of ≤6 months, or those who did not give an informed consent for the participation in the study. In the set of 582 hospitalizations included in the analysis, 494 unique patients were identified. Medical history was taken in all patients on admission by a qualified physician. Data included information on thromboembolic and cardiovascular risk factors.

### Diagnosis of atrial fibrillation

AF diagnosis was made with respect of the European Society of Cardiology Guidelines for the management of AF from 2010 and its update in 2012 [2, 3]. It was verified in all cases by two independent expert cardiologists, and required at least one episode of the AF recorded in a 12-lead electrocardiogram (ECG) made during the current hospitalization or an episode documented AF in a 12-lead ECG and/or 24-h ECG Holter monitoring in 6 months prior to the study enrollment. AF was defined as at least 30 s of an irregular heart rhythm without detectable P waves. Assessment of AF type was made basing on medical records. Paroxysmal AF was defined as a self-terminating episode, (usually within 48 h, which may continue for up to 7 days), whereas persistent AF was diagnosed when arrhythmia episode either lasted longer than 7 days or required termination by cardioversion, either with drugs or by direct current cardioversion. Permanent AF was diagnosed in the study population only if it was decided not to pursue rhythm control strategy.

### Assessment of thromboembolic and cardiovascular risk

Data on classical cardiovascular risk factors were collected and all patients had their stroke risk assessed in the CHADS_2_ and CHA_2_DS_2_-VASc scores according to the current scoring guidelines [4, 5]. In the CHADS_2_ score, one point was assigned for history of congestive heart failure, diabetes, hypertension, and age ≥75 years, whereas in the CHA_2_DS_2_-VASc score it was assigned for age between 65 and 74 years, female sex, history of hypertension, diabetes, heart failure, and vascular disease (history of myocardial infarction, presence of complex aortic plaque, or peripheral artery disease). Conditions linked with 2 point scores were history of stroke or transient ischemic attach (TIA) for CHADS_2_ score and history of stroke or TIA, and age ≥75 years in CHA_2_DS_2_-VASc score. Diagnosis of all factors implemented into the models was made basing on eligible medical records, taking prescription drugs applicable for the respective disease (i.e. hypoglycemic agents for diabetes), or as a de novo diagnosis according to the current diagnostic criteria.

### Echocardiographic evaluation

As soon as their clinical state was stable, all patients underwent a standard, full transthoracic echocardiography (TTE) according to the current standards of the European Association of Cardiovascular Imaging [11, 12]. All TTEs were performed using GE Vivid 7 Ultrasound Systems. Examinations were made and analyzed by two experienced echocardiographers. Standard views optimal for echocardiographic assessment were obtained. To avoid bias measurements were taken three times then averaged. The following parameters were measured for the purpose of the study: left ventricular end-diastolic diameter (LVEDd), left ventricular ejection fraction (LVEF), interventricular septal end-systolic diameter (IVSd), left ventricular posterior wall diameter (LVPWd) at end-diastole, left atrial diameter (LAd), and right ventricular diameter (RVEDd). End-diastole and end-systole were defined as the frame in the cardiac cycle in which the cardiac dimension was the largest and the smallest, respectively. LVEDd, IVSd, and LVPWd were measured from the parasternal long axis view using 2D echocardiography at the level of the mitral valve leaflet tips. LAd was measured using 2D obtained from the parasternal long axis view from the trailing edge of the posterior aortic wall to the leading edge of the posterior LA wall at the ventricular end-systole when the LA chamber is at its greatest dimension. Right ventricle was measured at the proximal portion of the RV outflow tract from the parasternal window at end-diastole using the long axis view. LVEF was calculated using the biplane method of discs (modified Simpson’s rule). Enlargement of the LA was classified into the four categories: normal, mildly enlarged, moderately enlarged, and severely enlarged in accordance with the European Society of Cardiology guidelines [11]. LA size was considered normal when LAd was <39 mm in women and <41 mm in men, otherwise it was considered enlarged. Mild enlargement was defined as LAd values 39–42 mm in women and 41–46 mm in men, moderate enlargement for 43–46 mm in women and 47–51 mm in men, and severe enlargement for ≥47 mm in women and ≥52 mm in men. Additionally, patients were divided according the LAd index, calculated as LAd/body surface area (calculated from Du Bois formula 0.007184 × weight^0.425^ × height^0.725^). LAd index-assessed atrial enlargement was classified as follows: normal LA—15–23 mm/m^2^, mildly enlarged LA—24–26 mm/m^2^, moderately enlarged—27–29 mm/m^2^, and severely enlarged as ≥30 mm/m^2^, both in men and women.

### Statistical analysis

Data were tested for normality using the Kolmogorov–Smirnov test. Continuous data are presented as mean and 95 % confidence intervals (CI), with statistical comparisons performed with the Mann–Whitney test or Student’s *t* test. For categorical variables comparison was made using either the *χ* ^2^ or Fisher exact tests. A *p* value of less than 0.05 was considered statistically significant, whereas the confidence intervals were 95 %. All statistical calculations were performed using commercially available SAS statistical software version 9.4 (SAS Institute, Inc., Cary, NC, USA).

## Results

The study population consisted of 494 consecutive patients (48.5 % male) at mean age of 73.4 ± 11.5 years. AF was classified as paroxysmal in 233 (47.3 %), as persistent in 109 (22.1 %), and as permanent in 151 (30.6 %) patients. As for the cardiovascular risk factors, hypertension was present in 387 (78.5 %) patients, diabetes mellitus in 164 (33.3 %) patients. Two hundred thirty-five patients (47.7 %) suffered from ischemic heart disease and 270 (54.8 %) were afflicted with heart failure. The population characteristics are shown in Table 1. Mean CHADS_2_ score was 2.5 ± 1.4 points while in CHA_2_DS_2_-VASc it was 4.3 ± 1.8.

**Table 1:** Baseline characteristics of the study population

Parameter	Value (*n* = 494)
Age (years)	73.4 ± 11.5
Male	239 (48.5 %)
Paroxysmal AF	233 (47.3 %)
Persistent AF	109 (22.1 %)
Permanent AF	151 (30.6 %)
Hypertension	387 (78.5 %)
Ischemic heart disease	235 (47.7 %)
Asthma or COPD	81 (16.4 %)
Heart failure	270 (54.8 %)
Diabetes mellitus	164 (33.3 %)
Prior stroke or TIA	88 (17.8 %)
Weight (kg)	78.2 ± 19.3
Height (cm)	166.3 ± 10.1
BMI (kg/m^2^)	28.8 ± 6.5
Heart rate on admission (bpm)	102.9 ± 34.4
Heart rate on discharge (bpm)	73.5 ± 13.5
LVEF (%)	50.3 ± 11.7
LVEDd (mm)	50.3 ± 7.2
IVDs (mm)	12.8 ± 2.1
LWPWd (mm)	12.2 ± 1.8
RVEDd (mm)	31.0 ± 5.2
LAd (mm)	47.7 ± 7.6
LA enlargement—total	425 (86.2 %)
LA mildly enlarged	99 (20.0 %)
LA moderately enlarged	130 (26.3 %)
LA severely enlarged	196 (39.7 %)
Mean CHADS_2_	2.5 ± 1.4
Mean CHA_2_DS_2_-VASc	4.3 ± 1.8

Values are mean ± SD or *n*(%)

*AF* atrial fibrillation, *BMI* body mass index, *bpm* beats per minute, *COPD* chronic obstructive pulmonary disease, *SD* standard deviation, *TIA* transient ischemic attack, *LVEDd* left ventricular end-diastolic diameter, *LVEF* left ventricular ejection fraction, *IVSd* interventricular septal end-systolic diameter, *LWPWd* left ventricular posterior wall diastolic diameter, *LAd* left atrial diameter, *RVEDd* right ventricular diameter

Analysis of the echocardiographic parameters revealed that LA was enlarged in 426 (86.2 %) patients. Details of patients’ characteristics according to presence of LA enlargement are presented in Table 2. The enlargement was classified as mild in 99 (20.0 %) patients. As moderate in 130 (26.3 %) patients, and as severe in 196 (39.7 %) patients.

**Table 2:** Characteristics of the study population according to the presence of atrial enlargement

Parameter	Patients without LA enlargement (*n* = 68)	Patients with LA enlargement (*n* = 426)	*p* value
Age (years)	71.3 ± 13.7	73.7 ± 11.0	0.30
Male	35 (51.5 %)	204 (48.0 %)	0.68
Paroxysmal AF	52 (76.5 %)	181 (42.6 %)	**<0.0001**
Persistent AF	8 (11.8 %)	101 (23.8 %)	**0.04**
Permanent AF	8 (11.8 %)	143 (33.6 %)	**0.0005**
Hypertension	51 (75.0 %)	336 (79.1 %)	0.54
Ischemic heart disease	29 (42.6 %)	206 (48.5 %)	0.44
Asthma or COPD	11 (16.2 %)	70 (16.5 %)	0.90
Heart failure	19 (27.9 %)	251 (59.1 %)	**<0.0001**
Diabetes mellitus	17 (25.0 %)	147 (34.6 %)	0.16
Prior stroke or TIA	10 (14.7 %)	78 (18.4 %)	0.58
Height (cm)	164.9 ± 8.2	166.4 ± 10.3	0.48
Weight (kg)	68.7 ± 13.0	79.3 ± 19.6	**0.001**
BMI (kg/m^2^)	25.4 ± 4.6	29.2 ± 6.5	**0.001**
TSH (μIU/ml)	3.5 ± 14.0	2.1 ± 5.3	0.17
K^+^ (mmol/L)	4.3 ± 0.7	4.5 ± 0.6	0.10
Heart rate on admission (bpm)	102.3 ± 33.4	103.0 ± 34.6	0.94
Heart rate on discharge (bpm)	73.5 ± 11.8	73.5 ± 13.7	0.93
Number of risk factors	2.4 ± 1.5	3.5 ± 1.6	**<0.0001**
LVEF (%)	54.1 ± 9.0	49.7 ± 12.0	0.06
LVEDd (mm)	47.0 ± 5.3	50.7 ± 7.4	**0.01**
IVDs (mm)	12.4 ± 2.0	12.9 ± 2.1	0.26
PWd (mm)	12.2 ± 1.7	12.2 ± 1.8	0.77
RVEDd (mm)	28.8 ± 4.5	31.3 ± 5.3	**0.01**
LAd (mm)	37.5 ± 4.8	49.1 ± 6.7	**<0.0001**
Mean CHADS_2_	2.0 ± 1.5	2.6 ± 1.3	**0.0005**
Mean CHA_2_DS_2_-VASc	3.8 ± 2.0	4.4 ± 1.8	**0.02**

Bold values indicate statistical significance (*p* < 0.05)

Values are mean ± SD or *n* (%)

*AF* atrial fibrillation, *BMI* body mass index, *bpm* beats per minute, *COPD* chronic obstructive pulmonary disease, *SD* standard deviation, *TIA* transient ischemic attack, *LVEDd* left ventricular end-diastolic diameter, *LVEF* left ventricular ejection fraction, *IVSd* interventricular septal end-systolic diameter, *LWPWd* left ventricular posterior wall diastolic diameter, *LAd* left atrial diameter, *RVEDd* right ventricular diameter

Patients with enlarged LA more often had history of heart failure than patients with normal LA (59.1 vs. 27.9 %; *p* < 0.0001), and had higher weight and body mass index (79.3 ± 19.6 vs. 68.7 ± 13.0 kg; *p* = 0.002 and 29.2 ± 6.5 vs. 25.4 ± 4.6 kg/m^2^; *p* = 0.001, respectively). There were also differences regarding the type of arrhythmia. Paroxysmal AF was more often present (76.5 vs. 42.6 %; *p* < 0.0001) in the group with normal LA size, while persistent and permanent AF was more often in patients with LA enlargement then in those with normal LA size (23.8 vs. 11.8 %; *p* = 0.04 and 33.6 vs. 11.8 %; *p* = 0.0005, respectively). Patients with enlarged LA compared to normal LA-sized population tend to present higher values of LVEDd (50.7 ± 7.4 vs. 47.0 ± 5.3 mm; *p* = 0.01) and RVEDd (31.3 ± 5.3 vs. 28.8 ± 4.5 mm; *p* = 0.009). There was also a trend for lower LVEF in patients with enlarged LA (49.7 ± 12.0 vs. 54.1 ± 9.0 %; *p* = 0.06).

**Fig. 1:**
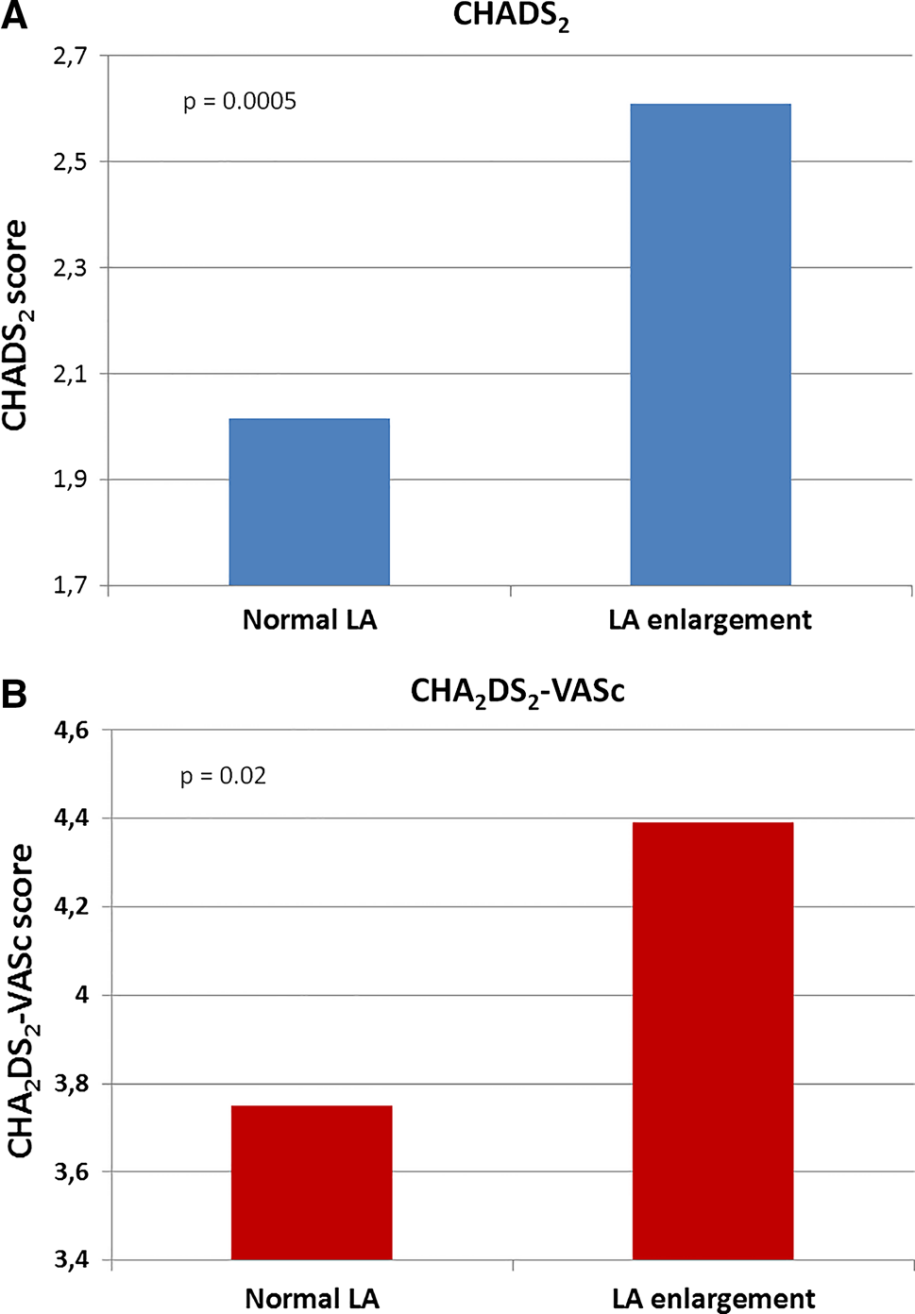
Mean **a** CHADS_2_ and **b** CHA_2_DS_2_-VASc scores in patients with and without left atrial enlargement. *Left atrial diameter criteria for enlargement* normal LA: ♀ < 39 mm, ♂ < 41 mm; mild LA enlargement: ♀ 39–42 mm, ♂ 41–46 mm; moderate LA enlargement: ♀ 43–46 mm, ♂ 47–51 mm; severe LA enlargement: ♀ ≥ 47 mm, ♂ ≥ 52 mm

**Fig. 2:**
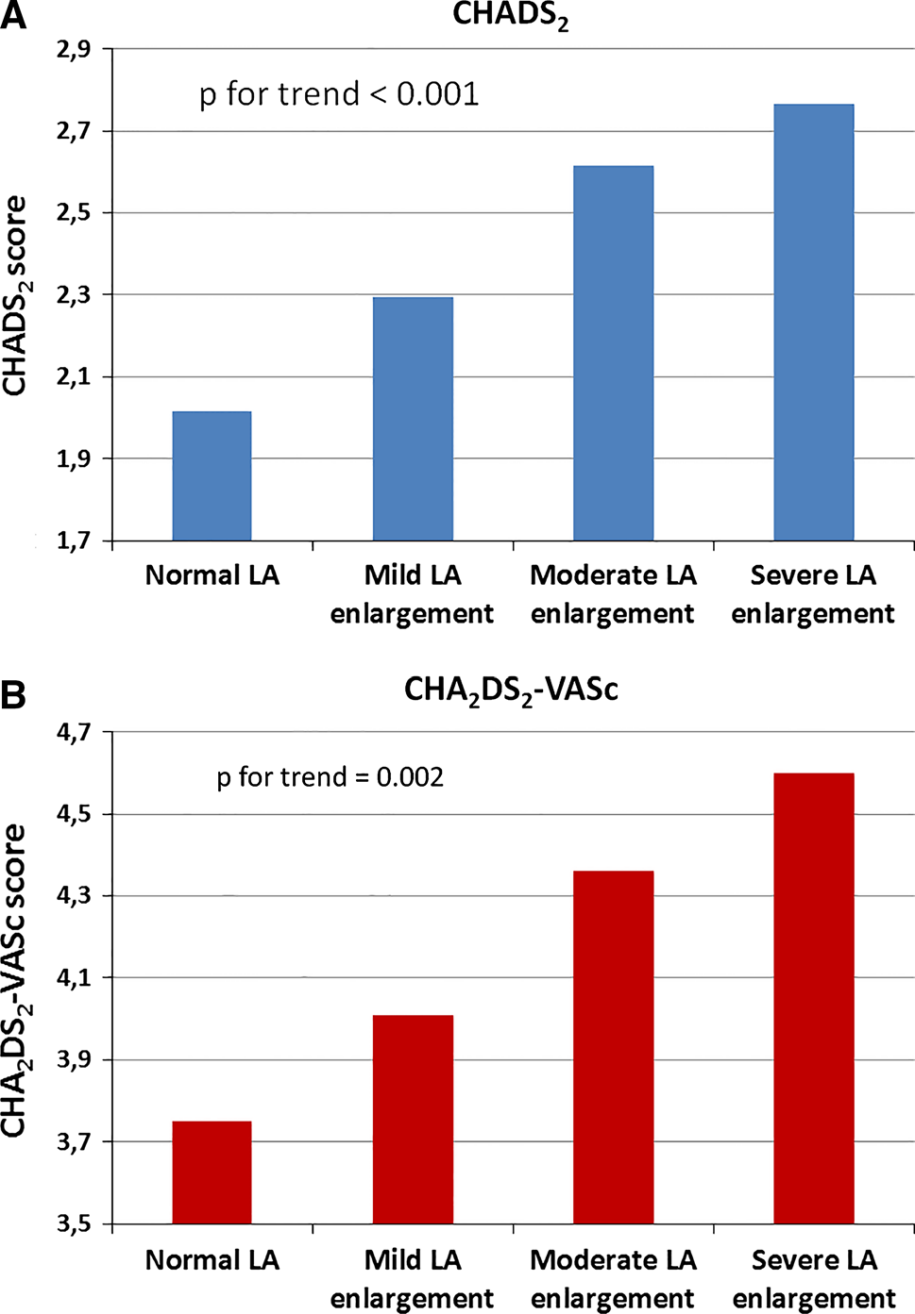
Relationship between CHADS_2_ (**a**) and CHA_2_DS_2_-VASc (**b**) scores and severity of the left atrium enlargement. *Left atrial diameter criteria for enlargement* normal LA: ♀ < 39 mm, ♂ < 41 mm; mild LA enlargement: ♀ 39–42 mm, ♂ 41–46 mm; moderate LA enlargement: ♀ 43–46 mm, ♂ 47–51 mm; severe LA enlargement: ♀ ≥ 47 mm, ♂ ≥ 52 mm

Thromboembolic risk assessed in CHADS_2_ and CHA_2_DS_2_-VASc scores, was higher in patients with LA enlargement, than without it. Figure 1a shows that patients with LA enlargement had higher mean CHADS_2_ score (2.0 ± 1.5 vs. 2.6 ± 1.3; *p* = 0.0005) than patients with normal LA, and the Fig. 1b illustrates that also CHA_2_DS_2_-VASc showed similar tendency (3.8 ± 2.0 vs. 4.4 ± 1.8; *p* = 0.02). After dividing patients into four categories: normal LAd, mildly, moderately, and severely enlarged LAd, we saw that along with the LA diameter also the risk in CHADS_2_ and CHA_2_DS_2_-VASc scores rose. For CHADS_2_ it was 2.0 ± 1.5 vs. 2.3 ± 1.3 vs. 2.6 ± 1.3 vs. 2.8 ± 1.4 (p for trend < 0.001), respectively (Fig. 2a) For CHA_2_DS_2_-VASc respective values were (3.8 ± 2.0 vs. 4.0 ± 1.7 vs. 4.4 ± 1.8 vs. 4.6 ± 1.8) (*p* for trend = 0.002) (Fig. 2b).

**Fig. 3:**
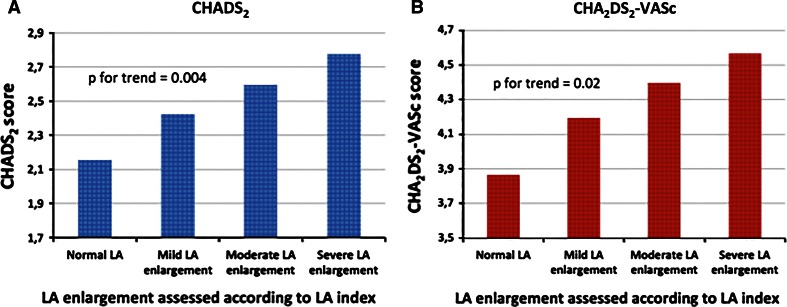
Relationship between CHADS_2_ (**a**) and CHA_2_DS_2_-VASc (**b**) scores and severity of the left atrium enlargement assessed by LAd index for body surface area. *Left atrial diameter index criteria for enlargement* normal LA: 15–23 mm/m^2^; mild LA enlargement: 24–26 mm/m^2^; moderate LA enlargement: 27–29 mm/m^2^; severe LA enlargement: ≥ 30 mm/m^2^

Similar trends were noticed, when LA enlargement was assessed basing on LAd index. Also in this case both mean CHADS2 (*p* = 0.004) and mean CHA2DS2-VASc (*p* = 0.02) values were the lowest in patients without LAd index-assessed LA enlargement, and the highest in patients with severe enlargement. Results of this subanalysis are depicted in Fig. 3a, b.

## Discussion

In the general population the prevalence of abnormal echocardiographic findings, including LA enlargement is high [13]. Echocardiographic abnormalities, especially associated with LA, are even more prevalent in AF patients [14]. Present study showed, that in a group of unselected AF patients, prevalence of LA enlargement is very high (up to 86 %). Interaction between the LA size and AF has been confirmed in many studies. Both conditions mutually exacerbate each other. In patients with diagnosed LA enlargement annual incidence of AF is higher than in patients with low LAd [15, 16]. On the other hand, in patients with AF, LA size tends to increase with the time course of disease. The trend is not very marked in patients with paroxysmal AF, but especially visible in those with the persistent form of the arrhythmia [17]. LA size cannot be attributable only to AF, in many cases its diameter could be a marker for associated vascular disease (especially untreated hypertension) and other comorbidities [18]. Nevertheless, LA size influences strongly also the clinical course of the arrhythmia, especially when the rhythm control strategy is pursued i.e. by cardioversion or ablation. Outcomes of those strategies are worse in patients with enlarged LA [19].

In the context of the present study, the most important issue is the association between LA dimension and elevated stroke risk. It was previously established that the atrial enlargement, measured by various echocardiographic parameters, in the general population is predictive of cardiovascular events including stroke, coronary artery disease, congestive heart failure, and fatal cardiovascular disease [20]. Atrial enlargement promotes turbulent blood flow and therefore formation of the thrombi in the atrium. Enlarged LA diameter (with addition of elevated thromboembolic risk in the CHADS_2_ score) is a predictor of incident LA thrombi [21]. As a consequence, patients with LA enlargement are at higher risk of stroke, and majority of the stroke type occurring in this group are cardioembolic strokes [22, 23].

In the present study we showed, that LA enlargement correlates with higher risk of stroke in both CHADS_2_ and CHA_2_DS_2_-VASc scores, more over the higher the LAd, the higher were the mean point scores. CHADS_2_ and CHA_2_DS_2_-VASc scores are widely used, simple risk assessment schemes, which showed to accurately predict the risk of stroke in AF patients [4, 24]. Other studies also showed them to be associated also with the risk of pulmonary embolism, or coronary artery disease severity [25, 26]. Nevertheless both schemes base only on medical history of patients, and do not incorporate any results from clinical tests. Therefore, they are prone to overlook some important issues, and underestimate thromboembolic risk, especially in patients in lower values range, which require anticoagulation therapy [6, 27]. LAd is a parameter the easiest to obtain, and routinely assessed in majority of AF patients, which, as showed in the current study may help identify patients at high risk of stroke. In many cases it does not require any additional measurement or indexation. Nevertheless, additional analysis in which LAd was indexed for body surface area was performed, showing similar results. LAd index is a parameter more and standardized than LAd, but harder to obtain without additional measurements and calculators [11].

Previously CHADS_2_ and CHA_2_DS_2_-VASc were also proven to be associated with echocardiographic risk factors for thromboembolism including LA smoke, sludge, thrombus, and slow LA appendage emptying velocity [28, 29]. Nevertheless, in some cases assessed by the scores as “low risk” we can still find LA thrombi. It was shown that approximately 8 % of patients with CHADS_2_ score of 0 or 1 have dense spontaneous echo contrast and 3 % have LA thrombi [30]. This proves that assessment of LA structure is as important as risk predicted by CHADS_2_ and CHA_2_DS_2_-VASc. Any additional tool, which will help properly assess the thromboembolic risk may be useful in the clinical practice, especially in the era, where many patients requiring anticoagulation do not receive such treatment [31, 32].

Current guidelines do not provide recommendations on the specific groups of patients in whom the TTE should be performed [2, 3]. The guidelines say that TTE can provide useful information to guide clinical decision-making, but do not recommend performing it in all AF patients. It can be due to limited availability or insufficient cost-effectiveness of the routine screening. Nevertheless growing body of literature suggest that TTE, can be useful and should be performed in all patients with low CHADS_2_ or CHA_2_DS_2_-VASc scores, who do not fulfill score-based criteria for introduction of anticoagulation treatment. In these patients any potentially pro-thrombotic abnormality should be an indication for introduction of the anticoagulation treatment. This kind of approach was already proven to be cost-effective and provide additional quality-adjusted life-years (QALYs), in a model where TTE was routinely performed in patients with low CHADS_2_ to score, and anticoagulants were introduced in case of TTE-detected left atrial abnormality [33]. LAd measurement is one of the easiest to obtain even by non-cardiologist in a routine TTE, it can provide additional benefit in risk stratification and help guide stroke prevention in the borderline risk groups.

The most important limitation of the study is the lack of other echocardiographic parameters concerning LA (including its volume or function) being assessed. Previous studies showed that most of the LA parameters, including i.e. area in four-chamber view, volumes by the ellipsoid, single- and biplane area-length formulas are strongly associated with echocardiographic markers of stroke risk, including LA thrombus, and are almost equally predictive [34]. In the present study, it was decided to assess the most widely obtained echocardiographic parameter concerning LA, being LAd. It was decided to include in the study this particular parameter, because of its clinical utility and being easy to obtain, but LA diameter may not accurately reflect LA size, because atrial dilatation can be eccentric. Nevertheless, LAd is the main parameter used in the atrial enlargement definition. Other parameters are, in many cases, limited to the clinical studies, and therefore not very useful in everyday practice. Simple, routinely measured LAd gives the clinicians additional, important information in the decision making process on thromboembolic risk.

In conclusion, in a large group of unselected non-valvular AF patients LA enlargement was highly prevalent. Presence of higher thromboembolic risk assessed by both CHADS_2_ and CHA_2_DS_2_-VASc scores was associated with LA enlargement. Echocardiographically assessed LA size may be an additional, easy to obtain parameter useful in thromboembolic risk stratification of AF patients.
